# Knowledge, attitudes, and practices in pediatric hypoglycemia in Italy (KAPPHy study)

**DOI:** 10.1007/s40618-026-02992-3

**Published:** 2026-07-22

**Authors:** Arianna Maiorana, Roberta Pajno, Francesco Tagliaferri, Alessandro Rossi, Enza Mozzillo, Maurizio Delvecchio, SIMMESN/SIEDP Intersocietary Working Group

**Affiliations:** 1 Division of Metabolic Diseases and Hepatology, Ospedale Pediatrico Bambino Gesù, Rome, Italy; 2 Pediatric Unit, IRCCS San Raffaele Institute, Milan, Italy; 3 Clinical Metabolic Reference Center, Fondazione IRCCS Ca’ Granda Ospedale Maggiore Policlinico, Milan, Italy; 4 Department of Clinical Sciences and Community Health, Dipartimento di Eccellenza, University of Milan, Milan, 2023-2027 Italy; 5 Department of Translational Medicine, Section of Pediatrics, University of Naples “Federico II”, Naples, Italy; 6 Department of Biotechnological and Applied Clinical Sciences, University of L’Aquila, L’Aquila, Italy

**Keywords:** Hypoglycemia, Children, Survey, SIMMESN, SIEDP

## Abstract

**Purpose:**

Childhood hypoglycemia is associated with relevant morbidity and likely long-term neurological sequelae. Diagnostic thresholds and management strategies remain quite heterogeneous, particularly beyond the neonatal period. We aimed to evaluate current diagnostic and therapeutic practices for pediatric hypoglycemia management in Italy among members of the Italian Society of Pediatric Endocrinology and Diabetology (SIEDP) and the Italian Society of Metabolic Diseases and Newborn Screening (SIMMESN).

**Methods:**

An anonymous electronic survey including 43 questions about management of pediatric hypoglycemia was sent to SIEDP and SIMMESN members. Descriptive statistics and comparative statistical analysis between respondents of the two societies were run.

**Results:**

A total of 118 respondents participated. Most of them reported direct clinical experience in pediatric hypoglycemia and routinely used critical sampling during hypoglycemic episodes. Diagnostic thresholds were heterogeneous: 34.7% reported 50 mg/dL, 33.1% 60 mg/dL, and 22.0% 70 mg/dL. Comparative analysis between societies showed no differences in most diagnostic procedures, but significant differences emerged in diagnostic thresholds (*p* = 0.005), with SIMMESN members selected 60 mg/dL more frequently (adjusted *p* = 0.020), and SIEDP members more frequently 70 mg/dL (adjusted *p* = 0.013). SIMMESN members reported more frequent use of genetic testing for etiological diagnosis. SIEDP respondents showed a frequent used of continuous glucose monitoring in the follow-up.

**Conclusion:**

Management of pediatric hypoglycemia in Italy is largely aligned with international recommendations. Clinically relevant variability persists, particularly in diagnostic thresholds, adoption of molecular analysis, and technologies, likely reflecting different clinical focus and patient populations. Our findings highlight the need for harmonized national recommendations and shared diagnostic definitions across scientific societies.

**Supplementary Information:**

The online version contains supplementary material available at https://doi.org/10.1007/s40618-026-02992-3.

## Introduction

Childhood hypoglycemia is a common problem that is associated with long-term neurological outcomes resulting from irreversible neuronal injury. Over the last two decades, several guidelines and recommendations have been proposed by influential scientific societies on neonatal hypoglycemia, including the American Academy of Pediatrics [1], the British Association of Perinatal Medicine [2], the European Foundation for the Care of the Newborn Infants [3], the Queensland Clinical Guidelines-Australia [4], the Canadian Pediatric Society [5] and the Pediatric Endocrine Society (PES) [6].

The recommendations made by the PES mainly address diagnosis and management of disorders causing recurrent or prolonged hypoglycemia that occurs in neonates beyond the first 72 h of life, infants and older children [6]. However, universally accepted guidelines for hypoglycemia are still lacking, and diagnostic and therapeutic strategies are heterogeneous. Indeed, a clear definition of hypoglycemia has not emerged and a threshold that allows normal neurological development has not been established [7].

Despite insufficient evidence to define neonatal hypoglycemia based on neurodevelopmental outcome alone, the neurological sequelae of neonatal hypoglycemia are well described [8–18]. Even mild neonatal hypoglycemia (< 2.6 mmol/l or < 47 mg/dl) in the neonatal period has been consistently associated with structural brain injury [19] and long-term neurodevelopmental impairment [20, 21]. Therefore, for the importance of timely recognition and appropriate management of neonatal hypoglycemia to prevent neurological disabilities, national guidelines exist in several countries [7].

In contrast, neurological outcomes related to hypoglycemic events occurring beyond the neonatal period have been reported more sporadically. Nevertheless, several studies describe significant neurodevelopmental sequelae in specific disorders characterized by recurrent hypoglycemia, such as congenital hyperinsulinism, which may present either in the neonatal period or later in infancy and childhood [22, 23].

Although recommendations and guidelines exist for the neonatal period, the scientific evidence in the pediatric age group remains quite limited. Prompted by this consideration, we aimed to evaluate how hypoglycemia is managed in infants and children in our country by the members of the two scientific societies, the Italian Society of Metabolic Diseases and Newborn Screening (SIMMESN) and the Italian Society of Pediatric Endocrinology and Diabetology (SIEDP), in comparison to PES recommendations [6]. The future goal will be to create Italian guidelines for the diagnosis and management of childhood hypoglycemia through a joint collaborative committee.

## Materials and methods

In September 2024, an intersociety study group involving the SIEDP and the SIMMESN was established. Within it, a hypoglycemia working group composed of three pediatric endocrinologists and three pediatric metabolic disease specialists has developed an anonymous electronic survey for members of the two Societies. The kick-off meeting was held on 7 October 2024, and after several meetings the final version of the survey was completed on 17 March 2025. The survey link was distributed by email to all society members on 12 May 2025. The survey was conducted between May 2025 and June 2025 using Google Forms (Google Inc., Menlo Park, CA, USA) and promoted via medical conferences, societies’ newsletters, and members’ mailing lists. It consisted of 43 questions in Italian, organized into four sections (Suppl. Box 1): (1) respondent characteristics and experience in managing childhood hypoglycemia; (2) organization of the respondent’s clinical center; (3) diagnostic practices for childhood hypoglycemia; and (4) management strategies for childhood hypoglycemia. Participants were informed that the survey focused on the diagnostic and therapeutic management of hypoglycemia due to genetic–metabolic disorders, or to endocrine causes, or to prolonged fasting or increased energy requirements. Diabetes-related hypoglycemia was outside the scope of the survey. Participation was open to all members of SIEDP and SIMMESN, and no restrictions were imposed regarding the number of respondents from a single institution.

### Statistical analysis

Results are presented as numbers and percentages. Comparative analyses were performed between respondents exclusively affiliated with the SIEDP and those exclusively affiliated with the SIMMESN, excluding members who reported membership in both societies. Differences between groups were assessed using the chi-square test; Fisher’s exact test and post-hoc analysis using pairwise proportion tests with Bonferroni correction were applied when appropriate. For comparisons involving ordinal variables derived from Likert-type scales, the overall distribution of response categories was compared between groups using the χ-square test for trend.

To identify independent determinants of the diagnostic glucose threshold selected, multinomial logistic regression analysis was performed using < 60 mg/dL (< 3.3 mmol/L) as the reference category. Independent variables included age, healthcare setting, professional profile, years of clinical experience, outpatient and inpatient management of hypoglycemia, number of patients managed in the last 5 years, and society affiliation (SIEDP vs. SIMMESN). Odds ratios (ORs) and 95% confidence intervals (CIs) were calculated. All tests were two-sided, and a p-value < 0.05 was considered statistically significant. Statistical analyses were performed using Python (pandas and SciPy libraries).

### Ethical consideration

The survey was reviewed and approved by the SIEDP/SIMMESN intersociety study group. As the survey was anonymous and did not collect personal data, formal ethics committee approval was not required. Participants were informed that their responses could be used for scientific publication.

## Results

### Characteristics of respondents

A total of 118 participants completed the survey. The response rate was 8.8% for SIEDP members and 8.5% for SIMMESN members. Table 1 summarizes their features. Most respondents had personally managed at least one infant or child with hypoglycemia (*n* = 113, 95.8%). The majority reported managing patients with hypoglycemia in outpatient settings (*n* = 97, 82.2%) as well as in inpatient settings (*n* = 109, 92.4%).

When considering clinical activity during the last five years, 60 respondents (50.8%) reported having followed between 10 and 50 children with hypoglycemia. Thirty-four respondents (28.8%) reported fewer than 10 patients, 16 (13.6%) between 50 and 100 patients, and 8 (6.8%) more than 100 cases.

**Table 1:** Characteristics of survey respondents

Characteristic	*n* (%)
Total respondents	118 (100)
Membership	SIEDP *n* = 76 (64.4%; response rate 8.8%)
	SIMMESN *n* = 35 (29.7%; response rate 8.5%)
	Both *n* = 7 (5.9%)
Age (years)	< 30 years: *n* = 2 (1.7%)
	30–40 years: *n* = 40 (33.9%)
	40–50 years: *n* = 39 (33.1%)
	> 50 years: *n* = 37 (31.4%)
Work setting	Research institute/University hospital *n* = 50 (42.4%)
	Tertiary care hospital *n* = 35 (29.7%)
	Secondary care hospital *n* = 20 (16.9%)
	Primary care hospital *n* = 10 (8.5%)
	Other work settings *n* = 3 (2.5%)
Discipline	Pediatricians *n* = 109 (92.4%)
	Other medical specialists *n* = 3 (2.5%)
	Residents *n* = 3 (2.5%)
	Dietitians/Nutritionists *n* = 3 (2.5%)
Clinical experience < 3 years	< 3 years: *n* = 8 (6.8%)
	3–10 years: *n* = 33 (28.0%)
	10–20 years: *n* = 37 (31.4%)
	> 20 years: *n* = 40 (33.9%)

### Diagnostic approach

**Fig. 1:**
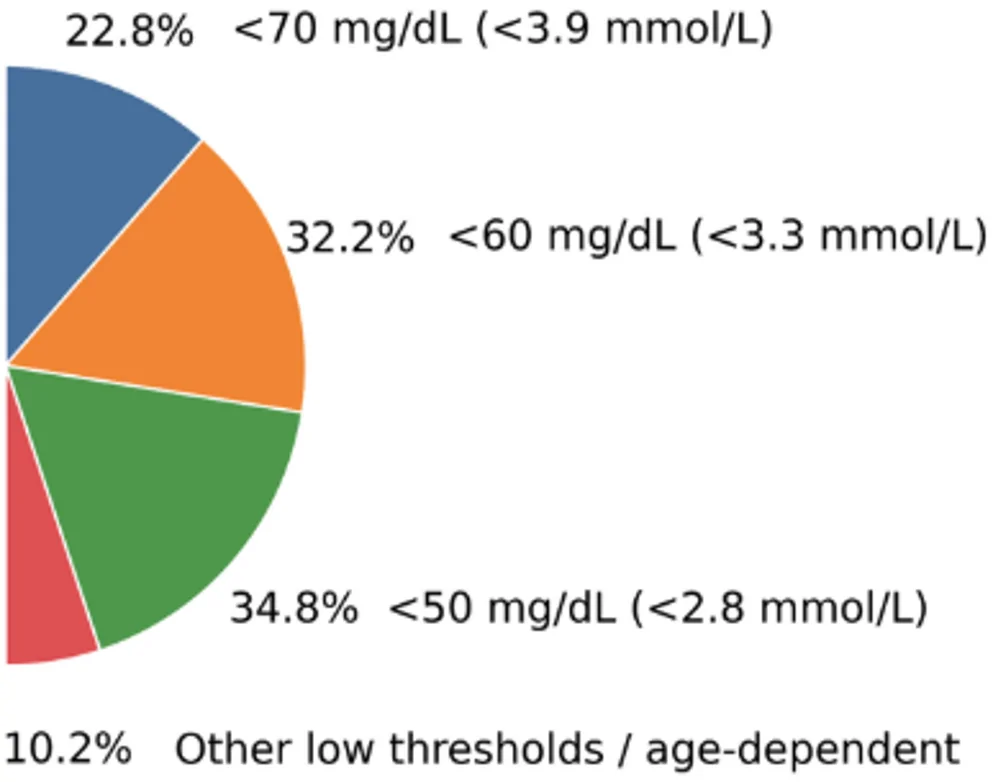
Thresholds used to define hypoglycemia in pediatric clinical practice. Distribution of glycemic thresholds reported by respondents for the definition of hypoglycemia in children. Other low threshold / age-dependent slice: 1 participant (0.8%): 45 mg/dl; 1 participant (0.8%): 55 mg/dl; 9 participants (8.5%): Depends on age

The diagnostic threshold for hypoglycemia was 50 mg/dl for 34.8%, 60 mg/dl for 32.2%, 70 mg/dl for 22.8% of participants or even different for the others (10.2%) (Fig. 1). Laboratory-measured plasma or serum glucose was the diagnostic method indicated by 46.6% of respondents, while 39.0% relied on point-of-care glucose measurements (glucometer or blood gas analysis), and 12.7% used the continuous glucose monitoring (CGM) for diagnostic purposes, whereas only 1.6% of clinicians indicated interchangeable use of multiple methods.

After ascertainment of hypoglycemia, collection of the critical sample is the major step for differential diagnosis. Almost all participants (98.3%) reported performing point-of-care biochemical assessments during hypoglycemia, while only 1.7% did not assess further analytes. The most frequently reported combination of additional point-of-care circulating analytes was lactate, ketone bodies, and ammonia together (40.7%). Measurement of ketone bodies alone was reported by 19.5%, while lactate combined with ketone bodies was assessed by 16.1%. Other combinations, including ammonia and selected hormonal parameters, were reported infrequently (≤ 2% each). Seventy-one-point 2% of respondents reported measuring a comprehensive hormonal and metabolic panel, including insulin, C-peptide, growth hormone, cortisol, and metabolic markers in the critical sample. A limited hormonal panel alone was reported by 17.8%, while 5.1% included additional investigations such as toxicological screening.

The collection of a critical sample is obtained always by 64 participants (54.2%) and often by 40 (33.9%). In contrast, 8 respondents (6.8%) reported collecting a critical sample sometimes and 6 (5.1%) rarely.

When a critical sample was not obtained during a spontaneous hypoglycemia, the supervised fasting test was used with variable frequency. Thirty-nine respondents (33.1%) reported performing it sometimes, 27 (22.9%) often, 24 (20.3%) rarely, and 22 (18.6%) always, whereas 6 (5.1%) reported never using this test. The glucagon stimulation test showed even greater heterogeneity: 35 respondents (29.7%) reported using it sometimes, 35 (29.7%) rarely, 18 (15.3%) often, 12 (10.2%) always, while 18 (15.3%) reported never performing it.

Genetic testing was also frequently incorporated into the etiological work-up. Forty-eight respondents (40.7%) reported performing genetic analyses often, 43 (36.4%) sometimes and 15 (12.7%) always, whereas 8 (6.8%) and 4 (3.4%) reported rare or no use, respectively. Approximately half of the respondents worked in centers that were able to perform genetic analyses for inherited disorders associated with hypoglycemia in-house (*n* = 61, 51.7%), while the remaining 57 respondents (48.3%) reported lacking this possibility. Among those indicating a preferred genetic testing method, gene panel analysis was most commonly used (*n* = 57, 48.3%), followed by whole-exome sequencing (*n* = 15, 12.7%); targeted single-gene analysis and whole-genome sequencing were each reported by 3 respondents (2.5%) (missing data *n* = 40).

CGM was widely adopted in the follow-up of children with hypoglycemia. Overall, 52 respondents (44.1%) reported using CGM often, 34 (28.8%) sometimes and 20 (16.9%) always; only 9 (7.6%) reported rare use and 3 (2.5%) reported not using CGM at all.

### Acute management

#### Without intravenous access

In the scenario of hypoglycemia of unknown etiology without intravenous access, the vast majority of respondents reported correcting the acute episode using oral glucose administration (*n* = 107, 90.7%). Additional strategies included fruit juice (*n* = 18, 15.3%), a carbohydrate-rich meal (*n* = 16, 13.6%) and other sugar-containing beverages (*n* = 9, 7.6%), often in combination.

More than half of respondents (*n* = 66, 55.9%) stated that their approach changes in the presence of a history of food refusal or selective eating, whereas 52 (44.1%) reported no change. When oral glucose was administered, the most common dosing strategy was the use of 1–3 sachets of 5 g sugar according to body weight (*n* = 73, 61.9%). Fixed weight-based doses were used by a smaller proportion of respondents: 23 (19.5%) reported using 200 mg/kg, 15 (12.7%) 300 mg/kg, and 6 (5.1%) 100 mg/kg.

Capillary glucose was usually rechecked within a short time frame. Specifically, 79 respondents (66.9%) reassessed glucose after 10 min, 21 (17.8%) after 5 min and 16 (13.6%) after 30 min; only 1 respondent (0.8%) reported rechecking after 60 min and 1 (0.8%) reported not routinely re-measuring glucose.

#### With intravenous access

When intravenous access was available, acute correction of hypoglycemia most consisted of a 10% dextrose bolus followed by continuous infusion at maintenance rate (*n* = 83, 70.3%). An isolated 10% dextrose bolus was reported by 22 respondents (18.6%), while 7 (5.9%) initiated a continuous infusion of 10% dextrose without a preceding bolus and 6 (5.1%) used a continuous infusion of 5% dextrose.

Among respondents who reported using an intravenous correction bolus (*n* = 104, missing data *n* = 14), 52 (50.0%) administered 200 mg/kg of glucose, 36 (34.6%) used 100 mg/kg, 13 (12.5%) used 300 mg/kg, and 3 (2.9%) reported doses greater than 300 mg/kg.

### Long-term pharmacological and nutritional strategies

In children with recurrent hypoglycemia of unknown etiology, no pharmacological treatments beyond glucose and nutritional measures were reported by 60 respondents (50.8%). Glucagon was used as adjunctive therapy by 42 respondents (35.6%), while 16 respondents (13.6%) reported the use of both glucagon and corticosteroids. None of the participants used corticosteroids by themselves.

With regard to the prevention of recurrence, 50.8% of respondents recommended the use of uncooked cornstarch or maltodextrins, 4.2% high-carbohydrates content diet, and 32.3% both. A small minority reported providing no specific preventive recommendations. In the context of intercurrent illness and/or poor appetite, 22.9% of respondents suggest oral rehydration solutions, 19.5% reported intensifying intake of uncooked cornstarch or maltodextrins, and 51.7% both.

### Comparison between SIEDP and SIMMESN members

Comparative analyses were performed between respondents exclusively affiliated with SIEDP (*n* = 76) and those exclusively affiliated with SIMMESN (*n* = 35). No significant differences emerged between groups with respect to the variables displayed in Table 1, but differences emerged in the hypoglycemia threshold, in the method used to assess glycemia for the diagnosis, in the frequency of use of genetic tests in the diagnostic process and advanced technology in the follow-up.

**Fig. 2:**
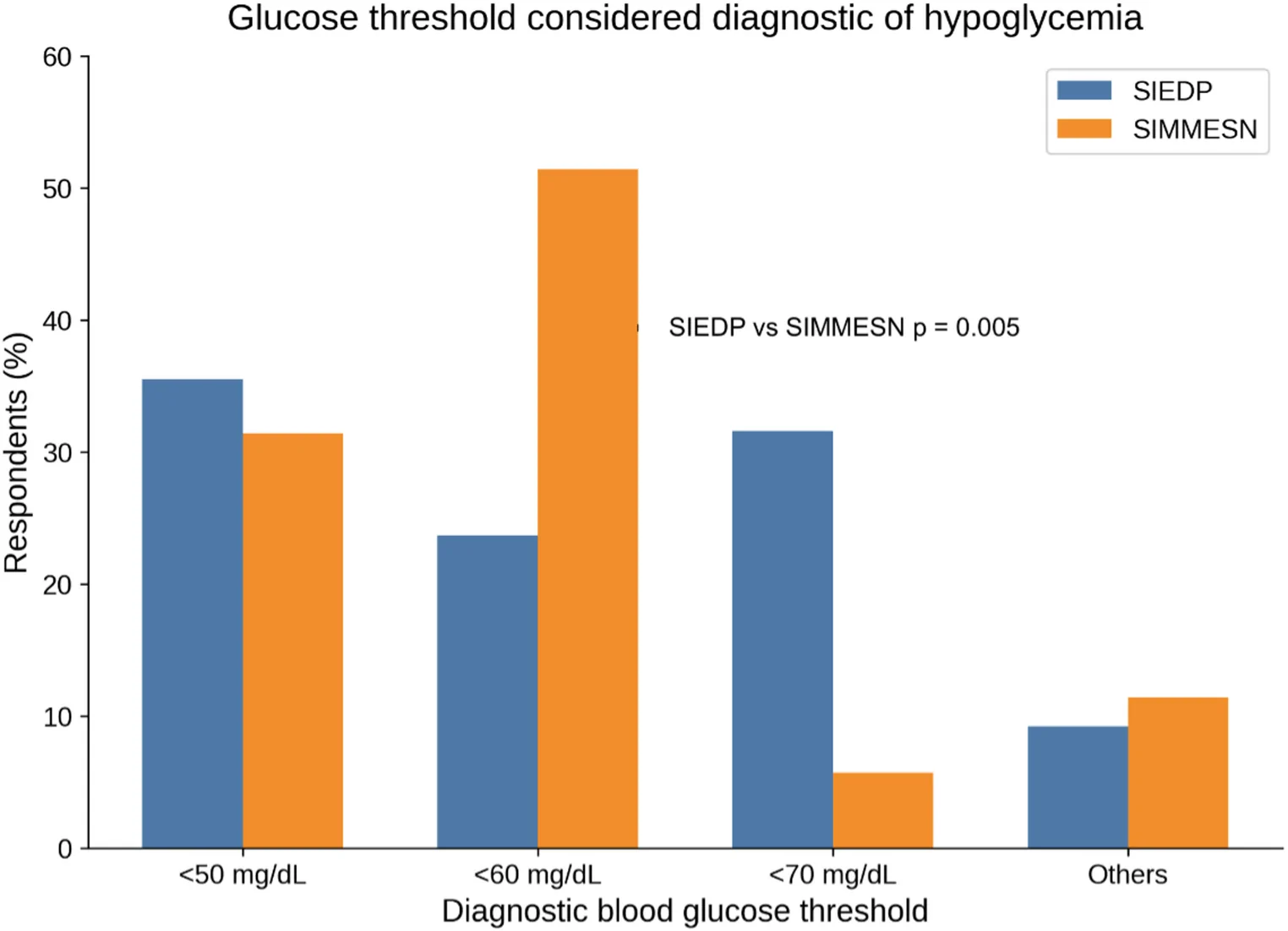
Diagnostic glucose thresholds for hypoglycemia according to scientific society. Comparison of diagnostic glucose thresholds for hypoglycemia as defined by the Italian Society for Pediatric Endocrinology and Diabetology (SIEDP) and the Italian Society of Metabolic Diseases and Newborn Screening (SIMMESN). Data are expressed as the percentage of respondents within each scientific society. The distribution of thresholds adopted was significantly different between societies (SIEDP vs. SIMMESN, *p* = 0.005). Post-hoc analysis demonstrated a higher prevalence of < 60 mg/dL threshold selection among SIMMESN members (adjusted *p* = 0.020), and a higher prevalence of < 70 mg/dL threshold selection among SIEDP members (adjusted *p* = 0.013)

A significant difference in the distribution of hypoglycemia diagnostic thresholds was observed between societies (*p* = 0.005). Post-hoc analysis demonstrated a higher prevalence of < 60 mg/dL threshold selection among SIMMESN members (adjusted *p* = 0.020), and a higher prevalence of < 70 mg/dL threshold among SIEDP members (adjusted *p* = 0.013). No between-group differences were found for lower thresholds or for alternative criteria (Fig. 2). On multinomial logistic regression analysis (reference category < 60 mg/dL), society affiliation and clinical experience were independently associated with the diagnostic glucose threshold selected. SIMMESN members had lower odds of reporting a threshold of < 50 mg/dL rather than < 60 mg/dL (OR 0.42, 95% CI 0.18–0.96, *p* = 0.041). Conversely, clinicians with more than 20 years of experience had higher odds of reporting a threshold of < 50 mg/dL compared with < 60 mg/dL (OR 2.87, 95% CI 1.03–8.01, *p* = 0.044) (Suppl. Table 1).

**Fig. 3:**
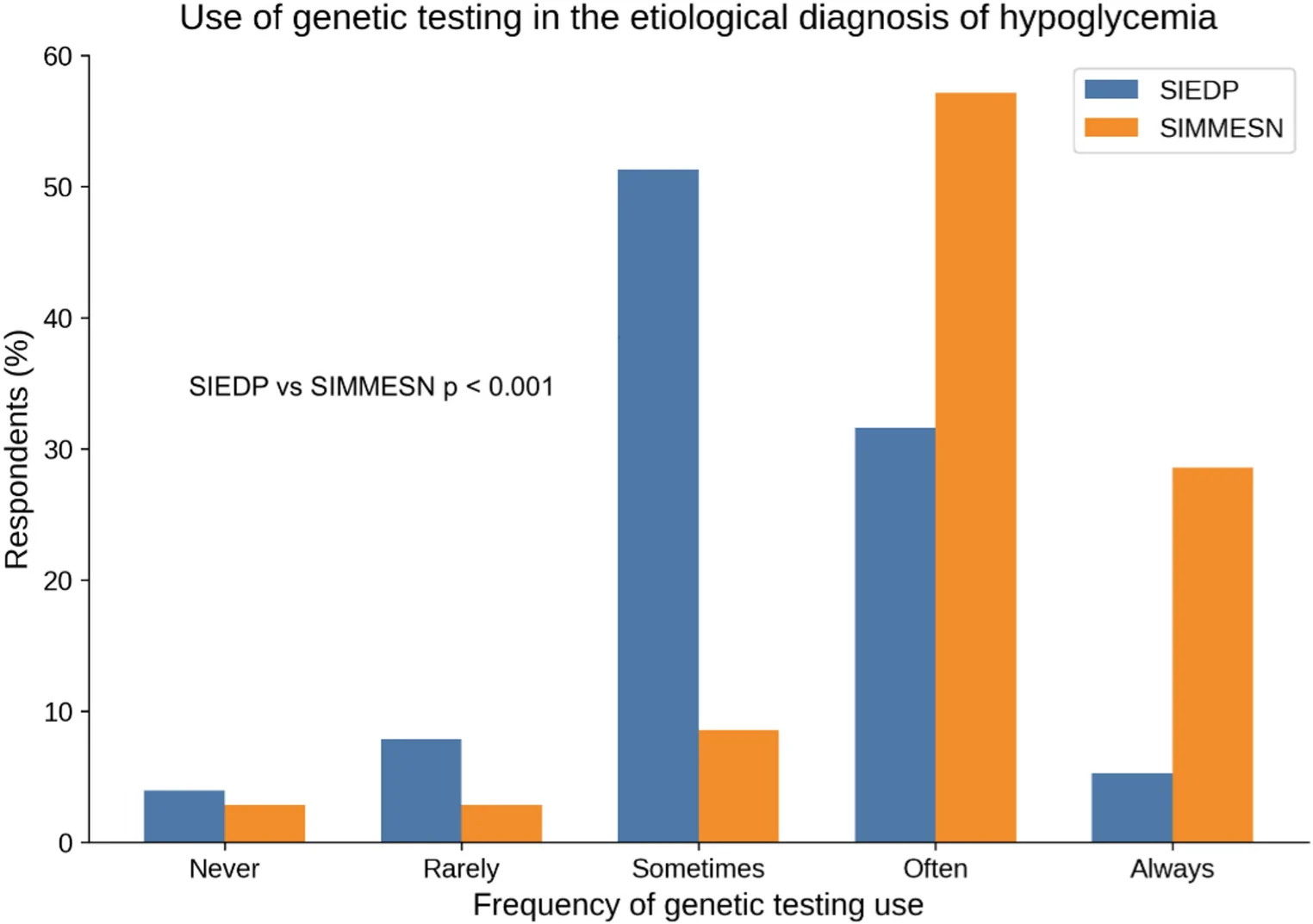
Use of genetic testing in the diagnostic evaluation of hypoglycemia according to scientific society. Frequency of genetic testing use among respondents from the Italian Society for Pediatric Endocrinology and Diabetology (SIEDP) and the Italian Society of Metabolic Diseases and Newborn Screening (SIMMESN), expressed as the percentage of respondents within each society across five categories. SIEDP respondents more frequently reported occasional use of genetic testing (“Sometimes”, *p* < 0.001), while SIMMESN respondents were more likely to report systematic use (“Always”, *p* = 0.003)

Furthermore, the use of genetic testing differed significantly between societies (*p* < 0.001). SIMMESN members responded more frequently “always” (OR 7.2, 95% CI 2.07–25.0, *p* = 0.001), whereas SIEDP members responded more frequently “sometimes” (OR 11.24, 95% CI 1.27–6.61, *p* < 0.001). No significant differences were observed in the remaining categories (Fig. 3).

**Fig. 4:**
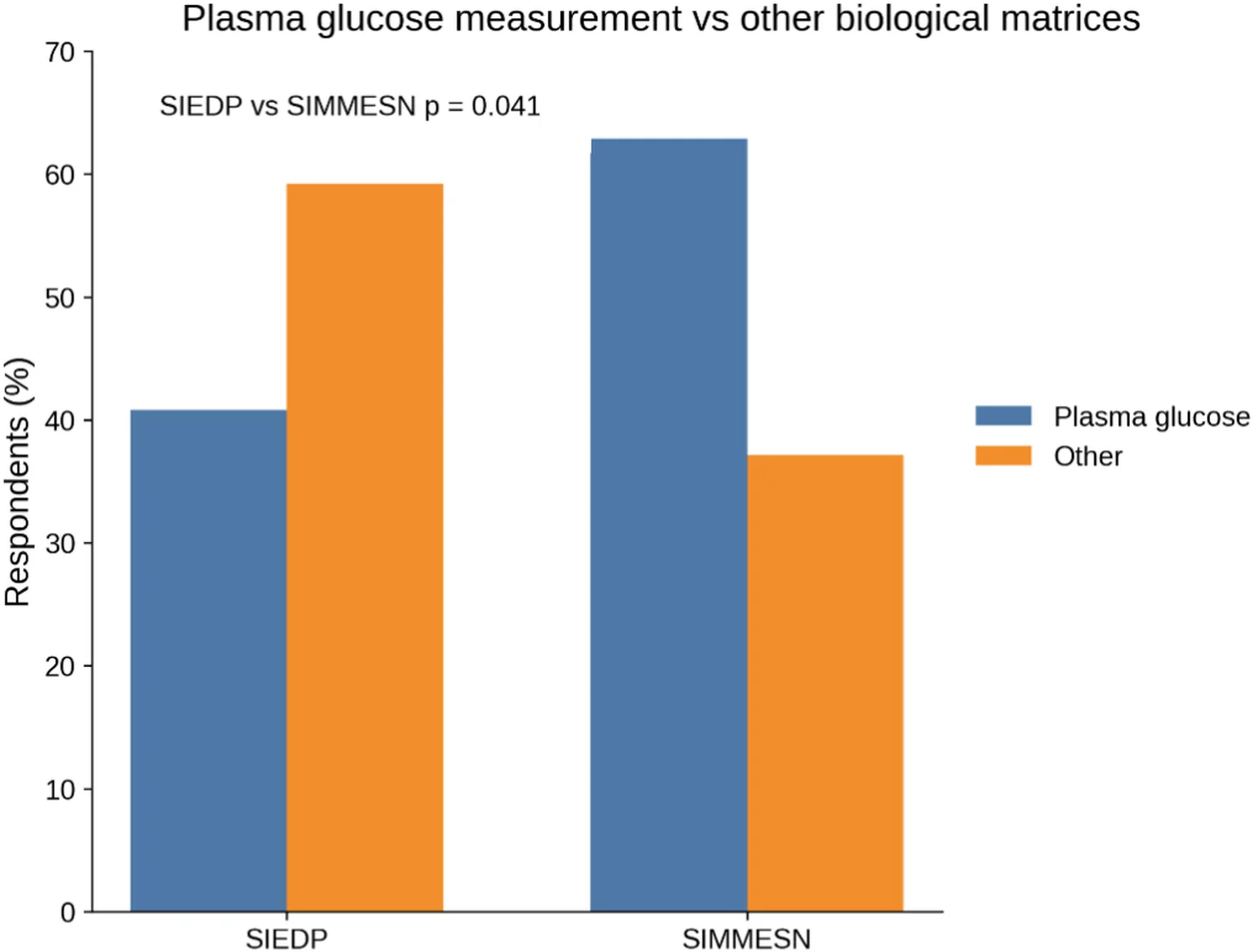
Diagnostic approach for blood glucose assessment for hypoglycemia diagnosis by scientific society. Distribution of diagnostic approaches for blood glucose assessment among Society for Pediatric Endocrinology and Diabetology (SIEDP) and Italian Society of Metabolic Diseases and Newborn Screening (SIMMESN) members after grouping responses into laboratory-based measurement and other approaches. Laboratory-based glucose measurement was more frequently reported by SIMMESN members than by SIEDP members

Among SIEDP members, laboratory-based plasma/serum glucose measurement and point-of-care testing (glucometer or blood gas analysis) were reported with similar frequency (40.8% each), while 18.4% indicated the use of CGM. In contrast, SIMMESN members more frequently reported relying on laboratory-based plasma glucose measurement (62.9%), followed by point-of-care testing (28.6%) (CGM, context-dependent combination of methods, and all available methods only 2.9% each). Overall, the distribution of diagnostic approaches differed significantly between the two societies (*p* = 0.017). Grouping responses into laboratory-based measurement versus other approaches confirmed the difference between groups (*p* = 0.041) (Fig. 4).

**Fig. 5:**
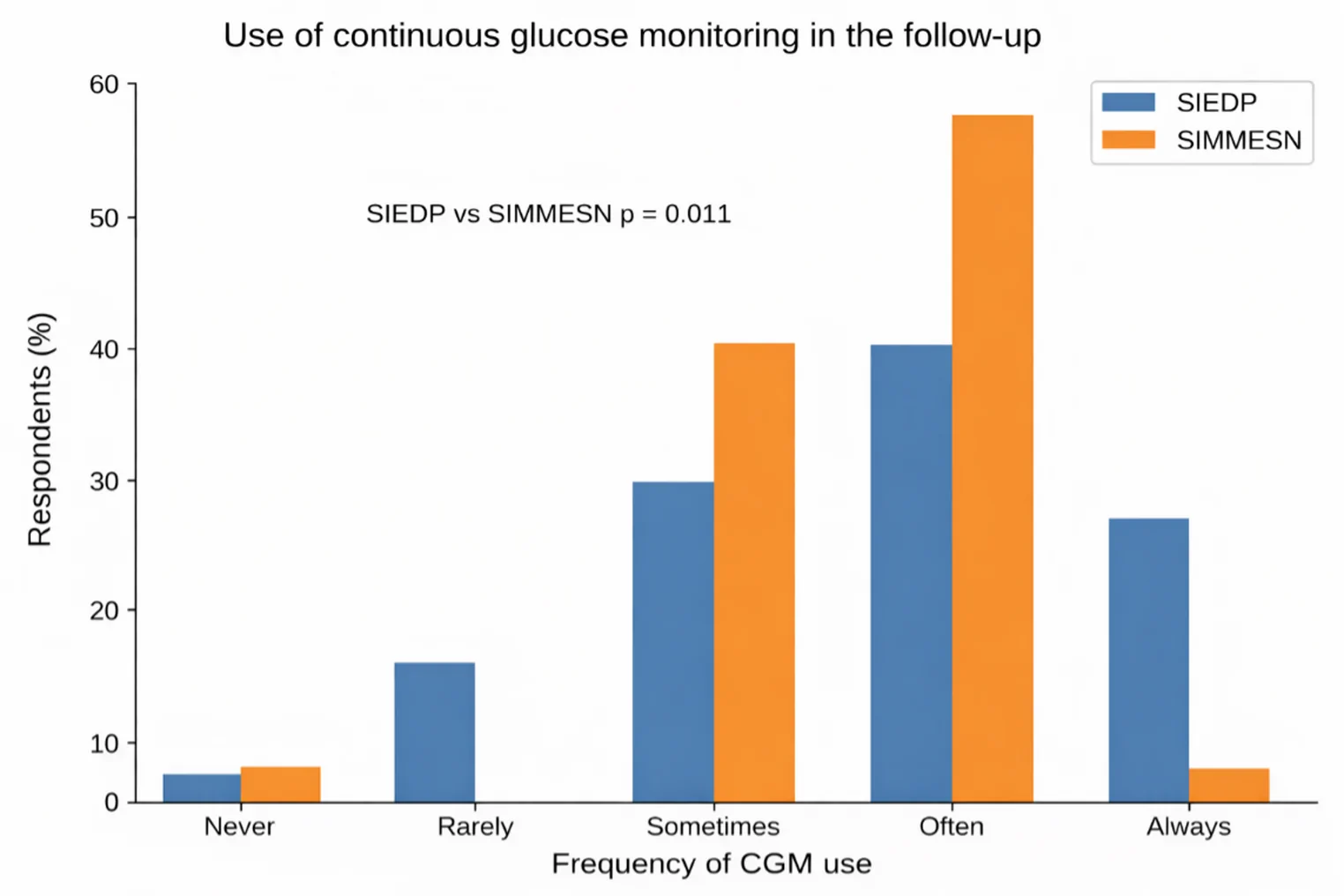
Use of continuous glucose monitoring (CGM) by scientific society. Frequency of CGM use in the follow-up of children with hypoglycemia among respondents affiliated with the Italian Society for Pediatric Endocrinology and Diabetology (SIEDP) and the Italian Society of Metabolic Diseases and Newborn Screening (SIMMESN), reported as the percentage of respondents within each society across five categories. The distribution of CGM use differed significantly between societies (SIEDP vs. SIMMESN, *p* = 0.011). SIEDP respondents were significantly more likely than SIMMESN respondents to report consistent use of CGM (“Always”, *p* = 0.034)

Similarly, the use of CGM in the follow-up differed significantly between scientific societies (*p* = 0.011). SIEDP respondents were significantly more likely to report consistent use of CGM (“Always”, *p* = 0.034), whereas no significant differences were observed for the other frequency categories (Fig. 5).

No additional statistically significant differences were observed between the two societies.

## Discussion

The results of this survey provide a comprehensive overview of how the diagnostic and therapeutic management of infants and children with non-diabetic hypoglycemia were managed in clinical practice in Italy. The invitation was sent to SIMMESN and SIEDP members, as they routinely manage patients with hypoglycemic conditions across the country and therefore represent an accurate snapshot of the national landscape. Results were compared with the existing literature on childhood hypoglycemia, particularly to PES recommendations, which mainly address diagnosis and management of hypoglycemia in neonates beyond the first 72 h of life, infants and older children [6].

PES committee adopted the framework of the Grading of Recommendations Assessment, Development, and Evaluation (GRADE) Working Group [24], for evidence quality. Table 2 compares the main findings of the survey to PES recommendations. The reported practices appear largely consistent with the international recommendations [6], particularly for diagnostic evaluation and acute treatment, whereas substantial variability emerged in areas where recommendations are limited or condition specific. Furthermore, the comparison between the two societies members shows interesting differences in some aspects of management, particularly regarding the diagnostic threshold for hypoglycemia, the molecular analysis and the use of advanced technologies.

**Table 2:** Comparison between international recommendations [6] and real-life clinical practice in the management of pediatric hypoglycemia. The “adherence rate” has been reported as the rate of responses in agreement with PES recommendations

Domain	International recommendations (PES)	Real-life practice (present survey)	Adherence rate
Threshold for diagnosis of hypoglycemia	Suggested 60 mg/dl in spontaneous critical sample; recommended 50 mg/dl in fasting test	Heterogeneous threshold	32.2%
Critical sample	Recommended	Routinely collected by most clinicians	Always 54.2% Often 33.9%
Method to define hypoglycemia	Suggested plasma glucose concentration	Not used routinely	46.6%
Supervised fasting test	Recommended when critical sample unavailable	Used with variable frequency	Always 18.6% Often 22.9%
Glucagon test	Recommended only in selected cases	Not used routinely	Always 10.2% Often 15.3%
Glucocorticoid monotherapy	Discouraged	Not used	100%
Acute treatment with IV access	IV glucose bolus + infusion recommended	Predominantly used	70.3%
IV glucose bolus dose	Indicative dose ~ 200 mg/kg; limited standardization	Significant variability between societies	50.0%
Prevention of recurrence	General principles (avoidance of prolonged fasting, nutritional therapy)	Highly heterogeneous approaches	Uncooked cornstarch or maltodextrins: 50.8%; high-carbohydrates content diet: 4.2%; both: 32.3%; no specific preventive recommendations: 12.7% During intercurrent illness and/or poor appetite: oral rehydration solutions: 22.9%; intensifying intake of uncooked cornstarch or maltodextrins 19.5%; both: 51.7%

Going into more detail, PES “recommends” that children able to communicate their symptoms, should be evaluated if a Whipple’s triad is documented (GRADE 1++++, strong recommendation, high evidence). For infants and younger children, evaluation is “suggested” if plasma glucose concentrations are below the normal threshold for neurogenic responses (< 60 mg/dL, GRADE 2+++0, weak recommendation, moderate evidence) (6). About 33% of the survey participants considered 60 mg/dl as a diagnostic threshold for hypoglycemia, particularly SIMMESN members. Conversely, nearly 35% reported a diagnostic threshold of 50 mg/dl, especially respondents with more than 20 years of experience. However, the value of 50 mg/dl was considered by PES as a threshold at which to collect a critical sample at the end of fasting test, which is strongly recommended in the absence of a spontaneous critical sample. Of note, 22% of respondents identified 70 mg/dL (3.9 mmol/L) as a diagnostic threshold for hypoglycemia despite the survey specifically addressing non-diabetic hypoglycemia. While this value is generally considered an alert threshold in diabetes care, its selection in the present survey may reflect a precautionary clinical approach or differing interpretations of existing recommendations. This finding underscores the lack of consensus currently surrounding the definition of hypoglycemia beyond the neonatal period and highlights the need for harmonized guidance.

The diagnostic threshold was significantly different between SIEDP and SIMMESN members, highlighting heterogeneity in the biochemical definition among pediatric specialists, likely reflecting the diabetology-oriented training of many SIEDP members, in which a hypoglycemia threshold of 70 mg/dL is traditionally adopted [25]. Ultimately, the threshold for hypoglycemia is debated, especially for infants and young children. In the more recent guidelines for congenital hyperinsulinism, plasma glucose of 50 mg/dl was established as the threshold for collecting the critical sample for all ages [26].

In addition, different biological matrices (plasma, blood for point-of-care, interstitial fluid for CGM) were also reported for the definition of hypoglycemia from the participants, with SIMMESN members who use laboratory based-measurements more frequently than SIEDP members that often prefer other approaches. However, PES recommendations state that plasma glucose concentration should be used to define hypoglycemia as whole blood glucose values are 15% lower than plasma glucose values, and point-of-care accuracy is limited in the range of hypoglycemia [6]. The observed heterogeneity in both diagnostic thresholds and glucose measurement methods may have important clinical implications, as plasma glucose, point-of-care measurements, and CGM-derived values are not directly interchangeable. This variability may contribute to both over- and underdiagnosis of hypoglycemia and strongly support the need for shared definitions and harmonized recommendations across scientific societies.

With regard to the diagnostic work-up, the routine collection of a critical sample during hypoglycemic episodes was widely reported, consistent with PES recommendations. The use of a supervised fasting test when a critical sample is unavailable was also reported, albeit with variable frequency, reflecting adherence to recommendations combined with heterogeneous implementation [6, 27, 28].

The glucagon stimulation test, which is recommended only in selected clinical scenarios [6, 26, 29–31], was not used routinely. With regard to the use of glucocorticoids, which is discouraged by PES recommendations, it was suggested by less than 15% of the respondents, and only in association with glucagon.

SIMMESN members performed more often the genetic testing. This finding likely reflects differences in clinical focus and patient populations, with metabolic clinicians more often involved in the management of inherited disorders presenting with hypoglycemia, where early detection and etiological definition are particularly relevant for establishing the correct pharmacological and nutritional therapy [32].

With regard to the management, greater heterogeneity was observed in the use of advanced technologies. CGM was frequently employed by respondents from both societies, with SIEDP members reporting a more frequent use. While this practice is well supported by the International Society for Pediatric and Adolescent Diabetes (ISPAD) recommendations for children and adolescents with diabetes [33], guidance for non-diabetic hypoglycemia is lacking [18, 34, 35]. Therefore, the widespread adoption observed in this survey suggests an extension of CGM use beyond formal indications, likely driven by clinical need rather than standardized recommendations. Despite the lack of recommendations for routine use of CGM in non-diabetic hypoglycemia, a growing use of this technology has been observed lately among SIMMESN members. Indeed, its use in patients with Congenital Hyperinsulinism [36–38] and Glycogen Diseases is currently expanding worldwide [39, 40].

The heterogeneous use of fasting test, glucagon test, and CGM reflects the growing recognition that childhood hypoglycemia requires a dynamic phenotyping approach, integrating glucose trends, fasting tolerance, and counter-regulatory responses over time rather than reliance on isolated biochemical measurements alone [41]. Similarly, most respondents reported to include an extensive endocrinological and metabolic work-up into the diagnostic evaluation of hypoglycemia, in line with international reports and guidance [6, 42–44]. No differences were found between societies in fundamental diagnostic steps and in the acute management as well, suggesting a shared foundational framework across pediatric endocrine and metabolic practice.

Acute management strategies were generally consistent with international recommendations. Oral glucose was widely used in the absence of intravenous access, but only 12.7% of respondents used the fixed weight-based doses of 0.2 g/kg as suggested by the European Resuscitation Council (ERC) Guidelines 2025 [45]. The intravenous glucose bolus followed by 10% glucose maintenance infusion represented the preferred approach when venous access was available. Although PES recommendations [6] and ERC Guidelines [45] suggest doses around 200 mg/kg, marked variability in intravenous dosing was observed. SIMMESN respondents more frequently reported the use of a 200 mg/kg bolus, whereas SIEDP members more often selected alternative doses, but the difference was not statistically different after Fisher’s exact test (*p* = 0.057). The variability in treatment choices may be attributed to the different professional backgrounds of the two societies. SIMMESN members are well trained in the emergency management of glycogen storage diseases, fatty acid β-oxidation defects, and congenital hyperinsulinism, whereas SIEDP members are more accustomed to managing diabetic hypoglycemia with glucagon and hypoglycemia due to adrenal crisis with hydrocortisone and additional dextrose at a dose of 500 to 1000 mg/kg [46].

Preventive strategies for recurrent hypoglycemia displayed the highest degree of variability of response as well as in the literature [6, 30, 31]. Although avoidance of prolonged fasting and nutritional therapy [6], frequent meals, special diets, use of maltodextrins or uncooked cornstarch in selected cases [26, 35] are described, practical and standardized preventive protocols are lacking because preventive strategies depend on the underlying diagnosis. Consistent with this gap, respondents reported a range of preventive approaches, particularly during intercurrent illnesses or reduced oral intake, such as increased administration of uncooked cornstarch or maltodextrins or use of oral rehydration solutions or both.

This survey has some limitations. First, it is a survey-based study and captures respondents’ perceptions rather than real-world practices. Second, there may be a potential response bias, particularly due to the survey’s reliance on practices and self-reported experience. Furthermore, non-respondents may have less experience or interest in pediatric hypoglycemia, likely skewing the findings. Third, this is a respondents-based survey, not a center-based survey, and thus more clinicians joining the same center may have participated. This approach could be considered debatable as it may introduce a center-related clustering effect, but we intentionally planned this approach to figure clinicians’ approach rather than centers’ approach. However, the large number of participants strengthens its representation of the real-world Italian landscape.

## Conclusion

Taken together, these findings indicate that real-life management of childhood hypoglycemia follows international recommendations to a large extent. Some aspects of management show critical issues when compared with the international literature, particularly where recommendations are scarce or not comprehensive across clinical contexts. This variability highlights the need for more integrated and pragmatic guidance addressing hypoglycemia across the pediatric age spectrum and calls for appropriate training and development of national guidelines to make childhood hypoglycemia management more homogeneous and consistent with international recommendations.

## Supplementary Information

Below is the link to the electronic supplementary material.

Supplementary Material 1

Supplementary Material 2
