# Supplementary material for: Knowledge, attitudes, and practices in pediatric hypoglycemia in Italy (KAPPHy study)
Source: J Endocrinol Invest. 2026 Jul 22;49(11):3463–73. doi: 10.1007/s40618-026-02992-3 (PMC13722730; doi:10.1007/s40618-026-02992-3)
Supplement: Supplementary file 1 [file 40618_2026_2992_MOESM1_ESM.docx]

Suppl. table 1. Multinomial logistic regression analysis of factors associated with diagnostic glucose threshold (reference category: <60 mg/dL)**.** No significant associations were observed for age, healthcare setting, professional profile, inpatient or outpatient activity, or number of patients managed.

| **Comparison** | **Variable** | **Odds Ratio** | **95% CI** | **p-value** |
| --- | --- | --- | --- | --- |
| <50 vs <60 | Society (SIMMESN vs SIEDP) | 0.42 | 0.18–0.96 | 0.041 |
| <50 vs <60 | Experience (>20 vs ≤20 years) | 2.87 | 1.03–8.01 | 0.044 |
| <70 vs <60 | Society (SIMMESN vs SIEDP) | 1.91 | 0.88–4.15 | 0.101 |
| <70 vs <60 | Experience (>20 vs ≤20 years) | 0.61 | 0.24–1.56 | 0.303 |
| Depends vs <60 | Society (SIMMESN vs SIEDP) | 0.73 | 0.19–2.84 | 0.653 |
