# Supplementary material for: Knowledge, attitudes, and practices in pediatric hypoglycemia in Italy (KAPPHy study)
Source: J Endocrinol Invest. 2026 Jul 22;49(11):3463–73. doi: 10.1007/s40618-026-02992-3 (PMC13722730; doi:10.1007/s40618-026-02992-3)
Supplement: Supplementary file 2 [file 40618_2026_2992_MOESM2_ESM.docx]

**Box 1A. Questionnarie “Management of Hypoglycemia in Pediatric Age” – ENGLISH VERSION**

The Inter-society Working Group of SIEDP / SIMMESN invites all members of the two Scientific Societies to participate in this survey, which has been designed to evaluate the management of hypoglycemia in pediatric patients across the national territory.

**Background.** Hypoglycemia in pediatric age represents a significant cause of morbidity. It can be mainly classified into:

1. Hypoglycemia due to genetic–metabolic disorders
2. Hypoglycemia due to endocrine causes
3. Hypoglycemia secondary to prolonged fasting or increased energy requirements

The management of these children is generally carried out either by metabolic pediatricians affiliated with SIMMESN or by pediatric diabetologists affiliated with SIEDP, depending on local healthcare organization and on the clinical and biochemical characteristics of the patient.

**Aim of the survey.** To describe the management of children with hypoglycemia across Italy.

**How to participate.** You are invited to participate in this study by sharing your experience in the management of hypoglycemia. If you agree to participate, you will be asked to complete an online survey that will take approximately 10 minutes. Your responses will be completely anonymous. You are also encouraged to share the survey with your colleagues. We thank you in advance for your contribution to this survey.

**ELECTRONIC CONSENT**

Please select your choice below. By clicking “I agree”, you declare that:

- You have read the information provided above
- You voluntarily agree to participate
- You are at least 18 years of age
- You are aware that the results of this survey will be disseminated for scientific purposes

If you do not wish to participate in the study, please decline participation by clicking “I do not agree”.	☐ I agree	☐ I do not agree

**Section 1. Participant profile**

**1. Please indicate your age:**

*☐ < 30 years	☐* *30–40 years*	*☐ 40–50 years	☐* ***>****50 years*

**2. In which region do you work?**

**3. How would you classify the healthcare setting where you work?**

*☐ Level I hospital	☐ Level II hospital	☐ Level III hospital*

*☐ Research institute / University hospital	☐ Non-hospital territorial healthcare facility*

*☐ Other:*

**4. Describe your professional profile:**

*☐ Pediatrician	☐ Physician (non-pediatric specialty)	☐ Dietitian / Nutritionist*

*☐ Medical resident	☐ Nurse*

**5. Do you have a second specialty?**

*If yes, please specify:*

**6. How long have you been working in pediatric endocrinology–diabetology or metabolic diseases?**

*☐ < 3 years	☐ 3–10 years	☐ 10–20 years	☐ 20 years*

**7. Have you ever personally managed an infant or child with hypoglycemia?**

*☐ Yes	☐ No*

**8. Do you manage infants or children with hypoglycemia in an outpatient setting?**

*☐ Yes	☐ No*

**9. Do you manage infants or children with hypoglycemia during hospitalization?**

*☐ Yes	☐ No*

**10. Considering all children with hypoglycemia you have managed or currently follow for treatment or prevention, how many have you managed in the last 5 years?**

*☐ < 10 children	☐ 10–50 children	☐ 50–100 children	☐ 100 children*

**11. Are you a member of:**

*☐ SIMMESN	☐ SIEDP	☐ Both SIEDP and SIMMESN*

**Section 2.**

**12. From where are patients referred to your unit? (multiple answers allowed)**

*☐ Emergency Department	☐ Primary care pediatrician*

*☐ Neonatology	☐ Other:*

**13. Patients are referred to your unit for: (multiple answers allowed)**

*☐ Diagnostic work-up of hypoglycemia*

*☐ Therapeutic management and follow-up of congenital hyperinsulinism or metabolic diseases*

*☐ Other:*

**14. At what age are patients referred to your unit? (multiple answers allowed)**

*☐ Neonatal period	☐ After discharge from the neonatal unit*

*☐ Pediatric age	☐ Adulthood*

**15. Which forms of hypoglycemia are followed at your unit? (multiple answers allowed)**

*☐ Congenital hyperinsulinism	☐ Transient neonatal hyperinsulinism*

*☐ Metabolic disorders associated with hypoglycemia*

*☐ Idiopathic ketotic hypoglycemia of childhood	☐ Other:*

**16. Patient follow-up at your unit includes: (multiple answers allowed)**

*☐ Outpatient follow-up visits	☐ Periodic inpatient reassessment*

*☐ Periodic day-hospital reassessment	☐ Other:*

**17. During follow-up, up to what age do you follow patients? (multiple answers allowed)**

*☐ Up to 14 years	☐ Up to 18 years	☐ Also in adulthood*

**Section 3**

**18. Which elements do you mainly consider in diagnosing hypoglycemia in an infant?**

*☐ Whipple’s triad	☐ Blood glucose regardless of clinical signs/symptoms*

*☐ Clinical signs/symptoms regardless of blood glucose	☐ Other:*

**19. Which elements do you mainly consider in diagnosing hypoglycemia in a child?**

*☐ Whipple’s triad	☐ Blood glucose regardless of clinical signs/symptoms*

*☐ Clinical signs/symptoms regardless of blood glucose	☐ Other:*

**20. Which method do you use to assess glycemia for the diagnosis of hypoglycemia?**

*☐ Serum/plasma glucose measured in a central laboratory*

*☐ Point-of-care glucose (glucometer or blood gas analysis)*

*☐ Continuous glucose monitoring (CGM)	☐ Other:*

**21. Which glucose threshold do you consider diagnostic of hypoglycemia?**

*☐ <70 mg/dL (<3.9 mmol/L)	☐ <60 mg/dL (<3.3 mmol/L)*

*☐ <50 mg/dL (<2.8 mmol/L)	☐ Other:*

**22. In addition to plasma glucose measurement, do you assess other circulating point-of-care analytes during hypoglycemia?**

*☐ Yes (go to next question)	☐ No (skip next question)*

**23. If yes, which POINT-OF-CARE analytes do you assess? (multiple answers allowed)**

*☐ Lactate	☐ Blood/urinary ketones	☐ Ammonia	☐ Other:*

**24. In diagnosing a child with hypoglycemia, do you routinely analyze the critical sample (i.e., blood sample obtained during hypoglycemia)?**

*☐ Always	☐ Often	☐Sometimes	☐ Rarely	☐ Never*

**25. Which analytes do you measure on the critical sample? (multiple answers allowed)**

*☐ Hormones (insulin, C-peptide, GH, cortisol)*

*☐ Metabolites (acylcarnitines, plasma amino acids, free fatty acids, urinary organic acids)*

*☐ Oral hypoglycemic agents and toxicology screening	☐ Other:*

**26. Which analytes do you measure in-house on the critical sample? (multiple answers allowed)**

*☐ Hormones (insulin, C-peptide, GH, cortisol)*

*☐ Metabolites (acylcarnitines, plasma amino acids, free fatty acids, urinary organic acids)*

*☐ Oral hypoglycemic agents and toxicology screening	☐ Other:*

**27. If one or more analytes cannot be measured in-house, what is your institution’s policy?**

*☐ The analyses cannot be performed*

*☐ Samples are stored and later sent to an external laboratory	☐ Other:*

**28. If you do not collect the critical sample, do you perform a fasting test for the etiological diagnosis of hypoglycemia?**

*☐ Always	☐ Often	☐Sometimes	☐ Rarely	☐ Never*

**29. Do you perform a glucagon test for the etiological diagnosis of hypoglycemia?**

*☐ Always	☐ Often	☐Sometimes	☐ Rarely	☐ Never*

**30. Do you use continuous glucose monitoring devices in the follow-up of children with hypoglycemia?**

*☐ Always	☐ Often	☐Sometimes	☐ Rarely	☐ Never*

**31. Do you perform genetic testing in the etiological diagnosis of hypoglycemia?**

*☐ Always	☐ Often	☐Sometimes	☐ Rarely	☐ Never*

**32. Is in-house genetic testing for inherited hypoglycemia disorders available in your institution?**

*☐ Yes (go to next question)	☐ No (skip next question)*

**33. Which method do you preferentially use for genetic diagnostic confirmation?**

*☐ Gene panel analysis	☐ Targeted gene analysis*

*☐ Whole exome sequencing	☐ Whole genome sequencing*

**Section 4**

**34. In a patient with hypoglycemia of unknown etiology without IV access, how do you correct the acute event?**

*☐ Administration of a carbohydrate-based meal	☐ Oral glucose administration*

*☐ Fruit juice	☐ Other sugar-containing beverages*

**35. Does this approach change if there is a history of refusal of specific foods?**

*☐ Yes	☐ No*

**36. If you administer oral glucose, in which form? (multiple answers allowed)**

*☐ 10% glucose solution	☐ 33% glucose solution*

*☐ Sugar sachet	☐ Glucosprint/Fastup or equivalent products*

*☐ I do not administer oral glucose	☐ Other:*

**37. If you administer sugar, which dosage do you use (oral)?**

*☐ 1/2/3 sugar sachets (5 g each) according to body weight	☐ 100 mg/kg	☐ 200 mg/kg	☐ 300 mg/kg	☐ I do not administer oral glucose*

**38. After how long do you recheck blood glucose?**

*☐ 5 minutes	☐ 10 minutes	☐ 30 minutes	☐ 60 minutes*

*☐ I do not recheck*

**39. In a patient with hypoglycemia of unknown etiology with IV access, how do you correct the acute event?**

*☐ 10% glucose bolus*

*☐ 5% glucose continuous maintenance infusion*

*☐ 10% glucose continuous maintenance infusion*

*☐ 10% glucose bolus + maintenance infusion*

**40. If you use a correction bolus, which dosage do you administer (IV)?**

*☐ 100 mg/kg	☐ 200 mg/kg	☐ 300 mg/kg	☐ 300 mg/kg*

**41. In a patient with recurrent hypoglycemia of unknown etiology, which additional drugs do you use?**

*☐ Glucagon	☐ Glucocorticoids	☐ Both	☐ None*

**42. To prevent recurrence of hypoglycemia in patients with unknown etiology, which recommendations do you provide?**

*☐ High-carbohydrate diet*

*☐ Supplementation with uncooked cornstarch or maltodextrins in children <1 year of age*

*☐ Both	☐ None	☐ Other:*

**43. In patients with hypoglycemia of unknown etiology, which recommendations do you provide in case of poor oral intake and/or infectious episodes?**

*☐ No specific recommendations	☐ Use of oral rehydration solutions*

*☐ Increased administration of cornstarch or maltodextrins in children <1 year of age*

*☐ Both	☐ Other:*

**Box 1B. Questionario sul “Management delle Ipoglicemie in età pediatrica” – VERSIONE ITALIANA**

Il GdL intersocietario SIEDP / SIMMESN invita tutti gli iscritti delle 2 Società Scientifiche a partecipare a questa survey disegnata per valutare la gestione dell’ipoglicemia sul territorio nazionale in età pediatrica.

**Contesto:** L’ipoglicemia in età pediatrica è una causa importante di morbidità. Possiamo distinguere principalmente:

1. ipoglicemie da causa genetico-metabolica
2. ipoglicemie da causa endocrina
3. ipoglicemie secondarie a digiuno prolungato o da aumento del fabbisogno energetico.

La presa in carico di questi bambini avviene generalmente da parte del pediatra metabolista che afferisce alla SIMMESN o del pediatra diabetologo che afferisce alla SIEDP, a seconda dell’organizzazione assistenziale del territorio e delle caratteristiche cliniche e biochimiche del paziente.

**Obiettivo di questa survey:** Descrivere la gestione del bambino con ipoglicemia sul territorio italiano

**Come partecipare:** Sei invitato a partecipare a questo studio condividendo la tua esperienza sull'ipoglicemia. Se accetti di partecipare a questo studio, ti verrà chiesto di rispondere a un sondaggio online che richiederà circa 10 minuti. Le tue risposte saranno completamente anonime. Sei inoltre invitato a condividere il sondaggio con i tuoi colleghi! Ti ringraziamo in anticipo per il tuo contributo a questo sondaggio.

**CONSENSO ELETTRONICO:**
Ti preghiamo di selezionare la tua scelta qui sotto. Cliccando sul pulsante "accetto", dichiari che:

- Hai letto le informazioni sopra riportate
- Acconsenti volontariamente a partecipare
- Hai almeno 18 anni di età
- Sei consapevole che i risultati di questa survey saranno oggetto di divulgazione scientifica

Se non desideri partecipare allo studio, ti preghiamo di declinare la partecipazione cliccando sul pulsante "non accetto".

*☐*Accetto	*☐*Non accetto

**Prima parte: Profilo del partecipante**

1. **Per favore, indica la tua età:**

*☐****<*** *30 anni	☐30 – 40 anni	☐40 – 50 anni	☐> 50 anni*

2. **In quale regione lavori:**
3. **Come classificheresti la realtà assistenziale dove lavori?**

*☐ Ospedale di 1° livello	☐ Ospedale di 2° livello*

*☐ Ospedale di 3° livello	☐ Ente di ricerca / Ospedale Universitario*

*☐ Struttura territoriale non ospedaliera	☐ Altro*

4. **Descrivi il tuo profilo professionale:**

*☐ Medico Pediatra	☐ Medico (altra specialità diversa da Pediatria)*

*☐ Dietista / nutrizionista	☐ Medico Specializzando*

*☐ Infermiere*

5. **Hai una seconda specialità?**

*☐ Si (indicare quale)	☐ No*

6. **Da quanto tempo lavori nel settore endocrino-diabetologico o del metabolismo?**

*☐ < 3 anni*	*☐ 3 – 10 anni*	*☐ 10 – 20 anni*	*☐ > 20 anni*

7. **Hai mai gestito in prima persona un lattante o bambino con ipoglicemia in età pediatrica?**

*☐ Si	☐ No*

8. **Gestisci ambulatorialmente lattanti o bambini con ipoglicemia?**

*☐ Si	☐ No*

9. **Gestisci lattanti o bambini con ipoglicemia in regime di ricovero?**

*☐ Si	☐ No*

10. **Pensa a quanti bambini con ipoglicemia hai mai gestito e a quanti ne segui per la terapia o la prevenzione. Quanti sono negli ultimi 5 anni?**

*☐ < 10 bambini	☐ 10-50 bambini	☐ 50- 100 bambini	☐ >100 bambini*

11. **Sei un socio:**

*☐ SIMMESN	☐ SIEDP	☐ Sia SIEDP che SIMMESN*

**Seconda sezione - PAZIENTI**

12. **Da dove vengono inviati i pazienti presso la tua unità (sono possibili più risposte):**

*☐ Pronto soccorso	☐ Pediatra di base*

*☐ Neonatologia	☐ Altro (specificare)*

13. **I pazienti vengono inviati alla tua unità per (sono possibili più risposte):**

*☐ Inquadramento diagnostico in ipoglicemia*

*☐ Gestione terapeutica e follow-up iperinsulinismo congenito o malattia metabolica*

*☐ Altro (specificare)*

14. **I pazienti vengono inviati alla tua unità (età di invio) (sono possibili più risposte):**

*☐ In epoca neonatale	☐ Dopo la dimissione dalla neonatologia*

*☐ In età pediatrica	☐ In età adulta*

15. **Quali forme di ipoglicemia sono seguite presso la tua unità (sono possibili più risposte):**

*☐ Iperinsulinismo congenito*

*☐ Forme transitorie di iperinsulinismo del neonato*

*☐ Malattie metaboliche con ipoglicemia*

*☐ Ipoglicemia chetotica idiopatica dell’infanzia*

*☐ Altro (specificare)*

16. **Il follow-up dei pazienti presso la tua unità (sono possibili più risposte):**

*☐ Prevede controlli ambulatoriali*

*☐ Prevede ricoveri di rivalutazione periodica*

*☐ Prevede DH di rivalutazione periodica*

*☐ Altro (specificare)*

17. **Durante il follow-up, fino a che età segui i pazienti (sono possibili più risposte):**

*☐ Fino ai 14 anni*	*☐ Fino ai 18 anni*

*☐ Anche in età adulta*	*☐ Altro (specificare)*

**Terza sezione - DIAGNOSI**

18. **Quali elementi consideri soprattutto nella diagnosi di ipoglicemia in un lattante?**

*☐ Triade di Whipple*

*☐ Glicemia indipendentemente dai segni/sintomi clinici*

*☐ Segni/sintomi clinici indipendentemente dalla glicemia*

*☐ Altro (specificare)*

19. **Quali elementi consideri soprattutto nella diagnosi di ipoglicemia in un bambino?**

*☐ Triade di Whipple*

*☐ Glicemia indipendentemente dai segni/sintomi clinici*

*☐ Segni/sintomi clinici indipendentemente dalla glicemia*

*☐ Altro (specificare)*

20. **Quale metodo di valutazione impieghi per la diagnosi di ipoglicemia?**

*☐ Glucosio (siero/plasma) analizzato nel laboratorio centralizzato*

*☐ Glucosio da point-of-care (glucometro o emogasanalisi)*

*☐ Glucosio da monitoraggio continuo del glucosio (CGM)*

*☐ Altro (specificare)*

21. **Quale soglia di glicemia consideri diagnostica di ipoglicemia?**

*☐ <70 mg/dL (<3.9 mmol/L)	☐ <60 mg/dL (<3.3 mmol/L)*

*☐ <50 mg/dL (<2.8 mmol/L)	☐ Altro (specificare)*

22. **In aggiunta alla valutazione del glucosio plasmatico, valuti ulteriori analiti circolanti point-of-care in corso di ipoglicemia?**

*☐ Si (vai alla domanda successiva)	☐ No (salta la domanda successiva)*

23. **Se hai risposto si alla precedente domanda, quali analiti point-of-care valuti? (Possibili risposte multiple)**

*☐ Lattato	☐ Chetonemia / Chetonuria*

*☐ Ammonio	☐ Altro (specificare)*

24. **Nella diagnosi di un bambino con ipoglicemia analizzi routinariamente il critical sample, ossia il campione ottenuto in ipoglicemia?**

*☐ Sempre	☐ Spesso	☐ A volte	☐ Raramente	☐ Mai*

25. **Quali analiti dosi su critical sample? (possibili risposte multiple)**

*☐ Ormoni (insulina, peptide C, GH, cortisolo)*

*☐ Metaboliti (acilcarnitine, aminoacidi plasmatici, acidi grassi liberi, acidi organici urinari)*

*☐ Farmaci ipoglicemizzanti orali ed esame tossicologico*

*☐ Altro (specificare)*

26. **Quali analiti dosi su critical sample in-house? (possibili risposte multiple)**

*☐ Ormoni (insulina, peptide C, GH, cortisolo)*

*☐ Metaboliti (acilcarnitine, aminoacidi plasmatici, acidi grassi liberi, acidi organici urinari)*

*☐ Farmaci ipoglicemizzanti orali ed esame tossicologico*

*☐ Altro (specificare)*

27. **Nel caso uno o piu’ analiti non possano essere dosati in-house quale è la policy della tua Istituzione?**

*☐ Non è possibile effettuare le relative analisi*

*☐ I campioni vengono conservati e successivamente spediti presso un laboratorio esterno*

*☐ Altro (specificare)*

28. **Se non raccogli il critical sample, nella diagnosi eziologica di ipoglicemia in un bambino effettui il test del digiuno?**

*☐ Sempre	☐ Spesso	☐ A volte	☐ Raramente	☐ Mai*

29. **Nella diagnosi eziologica di ipoglicemia in un bambino effettui il test al glucagone?**

*☐ Sempre	☐ Spesso	☐ A volte	☐ Raramente	☐ Mai*

30. **Nel monitoraggio di un bambino con ipoglicemia impieghi dispositivi per il monitoraggio continuo del glucosio?**

*☐ Sempre	☐ Spesso	☐ A volte	☐ Raramente	☐ Mai*

31. **Nella diagnosi eziologica di ipoglicemia, effettui analisi genetiche?**

*☐ Sempre	☐ Spesso	☐ A volte	☐ Raramente	☐ Mai*

32. **Nella tua Istituzione è possibile effettuare in-house l’analisi genetica per disordini ereditari associati a ipoglicemia**

*☐ Si (vai alla domanda successiva)	☐ No (salta la domanda successiva)*

33. **Quale metodo utilizzi preferenzialmente per la conferma diagnostica genetica? (una sola risposta)**

*☐ Analisi di pannelli di geni	☐ Analisi mirata di gene target*

*☐ Whole exome sequencing	☐ Whole genome sequencing*

**Quarta sezione - TRATTAMENTO ACUTO E PREVENZIONE**

34. **In un paziente con ipoglicemia senza diagnosi eziologica, SENZA ACCESSO IV, come effettui la correzione dell’evento acuto?**

*☐ Somministrando pasto a base di carboidrati	☐ Somministrando glucosio per os*

*☐ Somministrando succo di frutta	☐ Somministrando altra bevanda zuccherata*

35. **Tale approccio cambia se hai un dato anamnestico di rifiuto di determinati alimenti?**

*☐ Si	☐ No*

36. **Se somministri glucosio per os, in che forma (possibili più risposte)?**

*☐ Soluzione glucosata 10%	☐ Soluzione glucosata 33%*

*☐ Bustina di zucchero	☐ Glucosprint/Fastup o prodotti equivalenti*

37. **Se somministri lo zucchero, che dosaggio usi (per os)?**

*☐ 100 mg/kg	☐ 200 mg/kg*

*☐ 300 mg/kg	☐ 1/2/3 bustine di zucchero da 5 gr in base al peso*

38. **Dopo quanto tempo ricontrolli il valore?**

*☐ 5 minuti	☐ 10 minuti*

*☐ 30 minuti	☐ 60 minuti*

*☐ Non ricontrollo*

39. **In un paziente con ipoglicemia senza diagnosi eziologica, CON ACCESSO IV, come effettui la correzione dell’evento acuto?**

*☐ Soluzione glucosata 10% in bolo*

*☐ Soluzione glucosata 5% in infusione continua di mantenimento*

*☐ Soluzione glucosata 10% in infusione continua di mantenimento*

*☐ Soluzione glucosata 10% bolo + infusione di mantenimento*

40. **Se utilizzi il bolo di correzione, che dosaggio utilizzi (ev)?**

*☐ 100 mg/kg	☐ 200 mg/kg	☐ 300 mg/kg	☐ > 300 mg/kg*

41. **In un paziente con ipoglicemia ricorrente senza diagnosi etiologica, quali altri farmaci utilizzi?**

*☐ Glucagone	☐ Glucocorticoidi*

*☐ Entrambi	☐ Nessuno*

42. **Per prevenire la ricorrenza di ipoglicemia in caso di ipoglicemia senza diagnosi eziologica, che indicazioni fornisci?**

*☐ Dieta iperglucidica*

*☐ Supplementazione di amido di mais crudo, o maltodestrine nel bambino < 1 anno*

*☐ Entrambi*

*☐ Nessuna*

*☐ Altro (campo libero)*

43. **In caso di ipoglicemia senza diagnosi eziologica, che indicazioni fornisci in caso di inappetenza e/o episodi infettivi?**

*☐ Nessuna indicazione*

*☐ Utilizzo di soluzioni reidratanti orali*

*☐ Aumento delle somministrazioni di maizena, o maltodestrine nel bambino < 1 anno*

*☐ Entrambi*

*☐ Altro (campo libero)*
